# Supplementary material for: Causal Relationship between Aging and Anorexia Nervosa: A White-Matter-Microstructure-Mediated Mendelian Randomization Analysis
Source: Biomedicines. 2024 Aug 16;12(8):1874. doi: 10.3390/biomedicines12081874 (PMC11351342; doi:10.3390/biomedicines12081874)
Supplement: Supplementary file 1 [file biomedicines-12-01874-s001.zip › Supplementary Figure/Figure S4/Singe SNP plot of MR analysis for TL to ubm-b-1989.pdf]

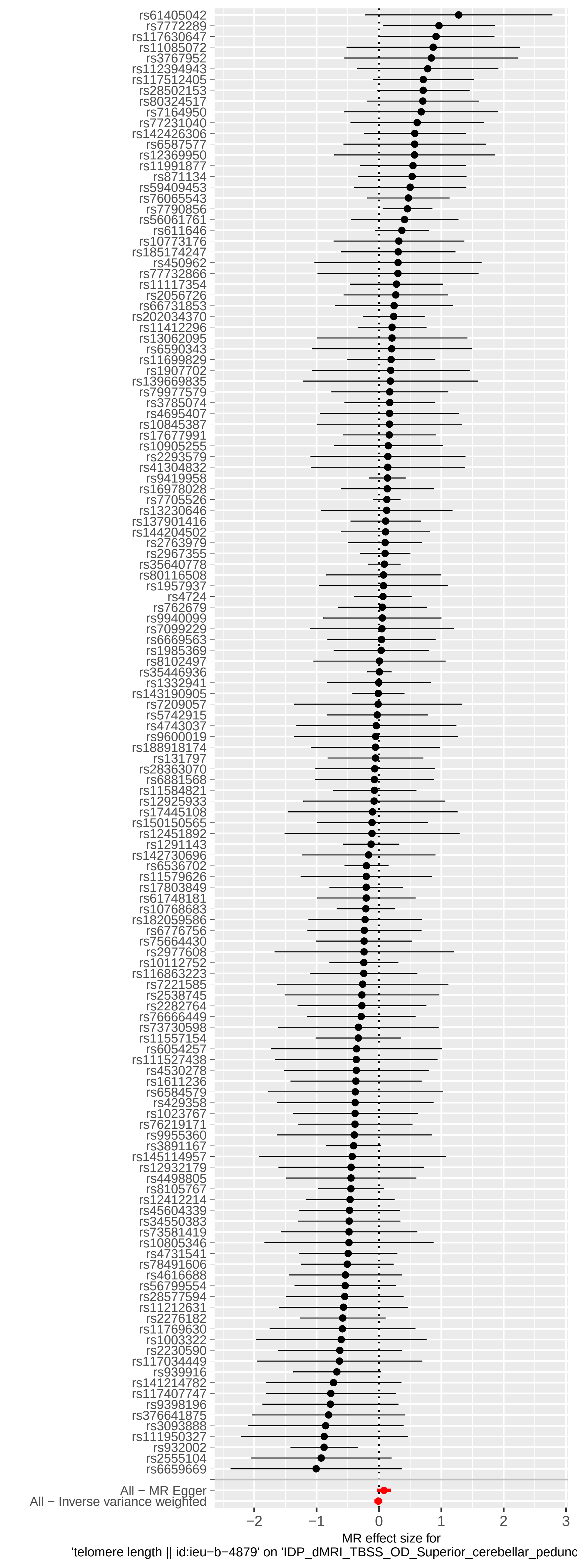
rs61405042
rs7772289
rs117630647
rs11085072
rs3767952
rs112394943
rs117512405
rs28502153
rs80324517
rs7164950
rs77231040
rs142426306
rs6587577
rs12369950
rs11991877
rs871134
rs59409453
rs76065543
rs7790856
rs56061761
rs611646
rs10773176
rs185174247
rs450962
rs77732866
rs11117354
rs2056726
rs66731853
rs202034370
rs11412296
rs13062095
rs6590343
rs11699829
rs1907702
rs139669835
rs79977579
rs3785074
rs4695407
rs10845387
rs17677991
rs10905255
rs2293579
rs41304832
rs9419958
rs16978028
rs7705526
rs13230646
rs137901416
rs144204502
rs2763979
rs2967355
rs35640778
rs80116508
rs1957937
rs4724
rs762679
rs9940099
rs7099229
rs6669563
rs1985369
rs8102497
rs35446936
rs1332941
rs143190905
rs7209057
rs5742915
rs4743037
rs9600019
rs188918174
rs131797
rs28363070
rs6881568
rs11584821
rs12925933
rs17445108
rs150150565
rs12451892
rs1291143
rs142730696
rs6536702
rs11579626
rs17803849
rs61748181
rs10768683
rs182059586
rs6776756
rs75664430
rs2977608
rs10112752
rs116863223
rs7221585
rs2538745
rs2282764
rs76666449
rs73730598
rs11557154
rs6054257
rs111527438
rs4530278
rs1611236
rs6584579
rs429358
rs1023767
rs76219171
rs9955360
rs3891167
rs145114957
rs12932179
rs4498805
rs8105767
rs12412214
rs45604339
rs34550383
rs73581419
rs10805346
rs4731541
rs78491606
rs4616688
rs56799554
rs28577594
rs11212631
rs2276182
rs11769630
rs1003322
rs2230590
rs117034449
rs939916
rs141214782
rs117407747
rs9398196
rs376641875
rs3093888
rs111950327
rs932002
rs2555104
rs6659669
All − MR Egger
All − Inverse variance weighted
−2
−1
0
1
2
3
MR effect size for
'telomere length || id:ieu−b−4879' on 'IDP_dMRI_TBSS_OD_Superior_cerebellar_pedunc
